# A simulation study of the strength of evidence in the recommendation of medications based on two trials with statistically significant results

**DOI:** 10.1371/journal.pone.0173184

**Published:** 2017-03-08

**Authors:** Don van Ravenzwaaij, John P. A. Ioannidis

**Affiliations:** 1 Department of Psychology, University of Groningen, Groningen, the Netherlands; 2 Department of Medicine, Stanford University, Stanford, California, United States of America; 3 Department of Health Research and Policy, Stanford University, Stanford, California, United States of America; 4 Department of Statistics and Meta-Research Innovation Center at Stanford (METRICS), Stanford University, Stanford, California, United States of America; National Taiwan University, TAIWAN

## Abstract

A typical rule that has been used for the endorsement of new medications by the Food and Drug Administration is to have two trials, each convincing on its own, demonstrating effectiveness. “Convincing” may be subjectively interpreted, but the use of p-values and the focus on statistical significance (in particular with *p* < .05 being coined significant) is pervasive in clinical research. Therefore, in this paper, we calculate with simulations what it means to have exactly two trials, each with *p* < .05, in terms of the actual strength of evidence quantified by Bayes factors. Our results show that different cases where two trials have a *p*-value below .05 have wildly differing Bayes factors. Bayes factors of at least 20 in favor of the alternative hypothesis are not necessarily achieved and they fail to be reached in a large proportion of cases, in particular when the true effect size is small (0.2 standard deviations) or zero. In a non-trivial number of cases, evidence actually points to the null hypothesis, in particular when the true effect size is zero, when the number of trials is large, and when the number of participants in both groups is low. We recommend use of Bayes factors as a routine tool to assess endorsement of new medications, because Bayes factors consistently quantify strength of evidence. Use of *p*-values may lead to paradoxical and spurious decision-making regarding the use of new medications.

## Introduction

Endorsement of medications (drugs and biologics) for clinical use is under rigorous control by regulatory agencies. Since 1962, the body that provides this control is the US Food and Drug Administration (FDA; [1]). The FDA has a critical function as the gateway for the adoption of new medications. The way the FDA endorses drugs and biologics is through clinical trials. New medications are tested, often against a placebo condition or an existing alternative, and statistical evidence is accumulated to quantify efficacy and to offer some reassurance of safety. By far the most common way to quantify evidence of efficacy is by defining a *null hypothesis* (e.g., the new medicine is just as effective as the placebo), collect data, and then generate using some statistical procedure what is known as a *p-value*. A *p*-value quantifies the probability of observing a difference between placebo and treatment at least as extreme as the difference observed in your data, given that the null hypothesis is true. In other words, if in reality there is *no* difference between the efficacy of the new medicine and the placebo, the probability of finding an effect at least as large as the one present in the data is equal to the size of the *p*-value. Typically, a *p*-value smaller than .05 is deemed *statistically significant*: it is considered sufficient evidence to *reject* the null hypothesis. Empirical studies have shown that the use of *p*-values is practically ubiquitous in biomedical research and this applies also to clinical trials [2].

Unfortunately, *p*-values are associated with a number of problems [3–7]. Firstly, it is impossible to quantify evidence *in favor* of the null hypothesis. A *p*-value can be used to reject or to fail-to-reject the null hypothesis, but never to accept it. Secondly, using p-values leads to over-rejection of the null hypothesis. The a-priori plausibility of the alternative hypothesis is not taken into account, as a result of which the alternative hypothesis gets endorsed if the null hypothesis is sufficiently unlikely. This leads to incorrect inference, in particular if the alternative hypothesis is even less likely. Thirdly, *p*-values are notoriously hard to interpret. Researchers generally want to use the data to infer something about their hypotheses, such as: what evidence do the data provide for the null hypothesis versus the alternative hypothesis? The *p*-value cannot answer these questions, instead giving an abstract number that quantifies the probability of obtaining a data pattern at least as extreme as the one observed *if* the null hypothesis were true. This definition proves to be very cryptic for most researchers in the field [8,9]. Finally, *p*-values do not allow for optional stopping, based on examining the preliminary evidence [10]. This means that a *p*-value can only be properly interpreted when the sample size for testing was determined beforehand and the statistical inference was carried out on the data of that exact sample size. In practice, additional participants are often tested when “the *p*-value approaches significance”, after which the *p*-value is calculated again. In clinical trials, this takes the form of interim analyses with the potential of early stopping at different points [11]. Alternatively, sometimes testing is discontinued when “an intermediate analysis fails to show a trend in the right direction”.

Another issue that is associated with the use of *p*-values is the almost ubiquitous focus on a .05 threshold (though some fields, like genomics, do employ more stringent criteria for significance [12]). Nevertheless, one trial may require a different degree of certainty than another [13]. The FDA recognizes the need for rigorous statistical evidence in their policy for drug endorsement. In their guidance for industry [14], the FDA states “With regard to quantity, it has been FDA’s position that Congress generally intended to require at least two adequate and well-controlled studies, each convincing on its own, to establish effectiveness.” (p. 3). “Convincing on its own” can be interpreted in many different ways and the meaning of “adequate and well-controlled” is somewhat subjective. However, given the widespread use of *p*-values and the strong emphasis on passing the threshold of *p* < .05, one may often interpret this guidance as meaning that two independent clinical trials with *p* < .05 are required before a new drug or biologic gets endorsed. Moreover, there is no specification of how many trials with *p*>.05 are allowed among the set of trials that contains these two statistically significant trials. Combining evidence in such a fashion is statistically inappropriate and can lead to wildly differing levels of *strength of evidence*.

In this paper, we will present through simulation the extent to which strength of evidence varies when employing a criterion for drug approval of having exactly two *p*-values lower than .05 for different scenarios. We focus on the scenario of exactly two statistically significant results, as this represents the FDA’s threshold for establishing effectiveness. We will show that in certain cases, this policy could actually lead to evidence in favor of the null hypothesis. We will quantify strength of evidence using Bayes factors [15,16]. A Bayes factor captures the relative evidence that the data provide for the alternative hypothesis against the null hypothesis in the form of an odds ratio. For example, when *BF* = 10, the data are 10 times more likely to have occurred under the alternative hypothesis than under the null hypothesis. On the other hand, when *BF* = 0.1, the data are 10 times more likely to have occurred under the null hypothesis than under the alternative hypothesis. As for interpreting the strength of evidence as quantified by a Bayes factor, a Bayes factor between 1 and 3 (or, conversely, between 1/3 and 1) is considered ‘not worth more than a bare mention’, a Bayes factor between 3 and 20 (or, conversely, between 1/20 and 1/3) is considered ‘positive’, and a Bayes factor between 20 and 150 (or, conversely, between 1/150 and 1/20) is considered ‘strong’ [17].

In the next section, we will describe the set-up of our simulations in detail. Then, we will present the results of our simulations, demonstrating both the range in strength of evidence and the proportion of times the evidence actually points in favor of the null hypothesis. We will conclude with a discussion of the implications of our results for regulatory assessment of new medications.

## Method

We conducted three sets of simulations. For every set, we generated 12,500 data sets. All of the data sets were intended to mimic two–condition between–subjects experiments with an experimental group and a control (e.g. placebo) group. A two-tailed *t*-test with a threshold of *p* < .05 combined with selection as “successes” only of those results for which all statistically significant effects are in the direction of the experimental arm being better than the control (e.g. placebo) arm. The three sets of simulations differed on the true population effect size between the two groups. In the first set of simulations, the true population effect size was small (0.2 standard deviations, or 0.2 SD), in the second set of simulations, the true population effect size was medium (0.5 SD), and in the third set of simulations, the true population effect size was zero (0 SD) [18]. Empirical evidence suggests that most effective treatments have small or modest effects, in particular when major clinical outcomes are concerned and very large effects are uncommon, except as chance findings in very small trials [19].

Therefore, our simulations are:

*p_123_* ~ *N*(0, 1)
*e_1_* ~ *N*(0.2, 1)
*e_2_* ~ *N*(0.5, 1)
*e_3_* ~ *N*(0, 1)

where *p*_*123*_ indicates simulated data for the placebo groups in all sets of simulations, and *e*_*1*_, *e*_*2*_, and *e*_*3*_ indicate simulated data for the experimental groups in the first, second, and third set of simulations respectively. The notation ∼*N*(,) indicates that values were drawn from a normal distribution with mean and standard deviation parameters given by the first and second number between parentheses, respectively.

For each effect size simulation set, we ran five different kinds of number of trial simulations: one with 2 trials with statistically significant results out of 2 performed, one with 2 significant results out of 3 performed, one with 2 significant results out of 4 performed, one with 2 significant results out of 5 performed, and one with 2 significant results out of 20 performed. This was achieved by continuously regenerating data until exactly 2 significant results emerged. Note that our simulations are not concerned with the likelihood of obtaining exactly 2 out of 5 significant results given a certain effect size. The purpose of our simulations is to demonstrate the range of strengths of evidence if such a scenario were to occur.

These simulations reflect different scenarios: on one end the scenario in which exactly two trials were conducted and both were statistically significant in the expected direction, on the other end the scenario in which twenty trials were conducted and exactly two were significant in the expected direction (and 18 were not statistically significant). We also varied the number of participants per group. We ran five conditions: n = 20, n = 50, n = 100, n = 500, and n = 1,000.

Thus, to sum up, our simulations varied along the following dimensions:

1. Effect size: small (0.2 SD), medium (0.5 SD), and zero (0 SD)
2. Number of total trials: 2, 3, 4, 5, and 20
3. Number of participants: 20, 50, 100, 500, and 1,000

This resulted in a total of 75 types of simulations. We replicated each simulation type 500 times. In addition to these simulations, we performed sensitivity analyses with simulations that used individual differences in the effect size distribution and unequal variance in the two groups (see S1 and S2 Files for details).

We calculated one-sided JZS Bayes factors for the combined data from the total number of trials conducted [20]. This one-sided Bayes factor quantifies the relative likelihood of the one-sided alternative hypothesis, the experimental group has a higher mean than the control group, against the null hypothesis, the experimental group has the same mean as the control group. Bayes factors were calculated using the BayesFactor package available in the statistical software package R (R package available at https://cran.r-project.org/web/packages/BayesFactor/index.html).

For each replication, we computed an independent-samples one-sided Bayesian *t*-test for the data of all trials combined. The JZS Bayes factor is calculated by comparing the marginal likelihood of the data under the null hypothesis to the marginal likelihood of the data under the alternative hypothesis, integrated over a range of plausible alternative hypotheses. The range of alternative hypotheses is given by a prior on the effect size parameter *δ*, which follows a Cauchy distribution with a width of *r* = √2/2 (see [20], Equation in note 4 on page 237). A mathematically equivalent way of obtaining the JZS Bayes factor, perhaps more intuitive, is to divide the height of the prior distribution by the height of the posterior distribution, evaluated at effect size *δ* = 0 (for a mathematical proof, see [21]).

## Results

**Fig 1:**
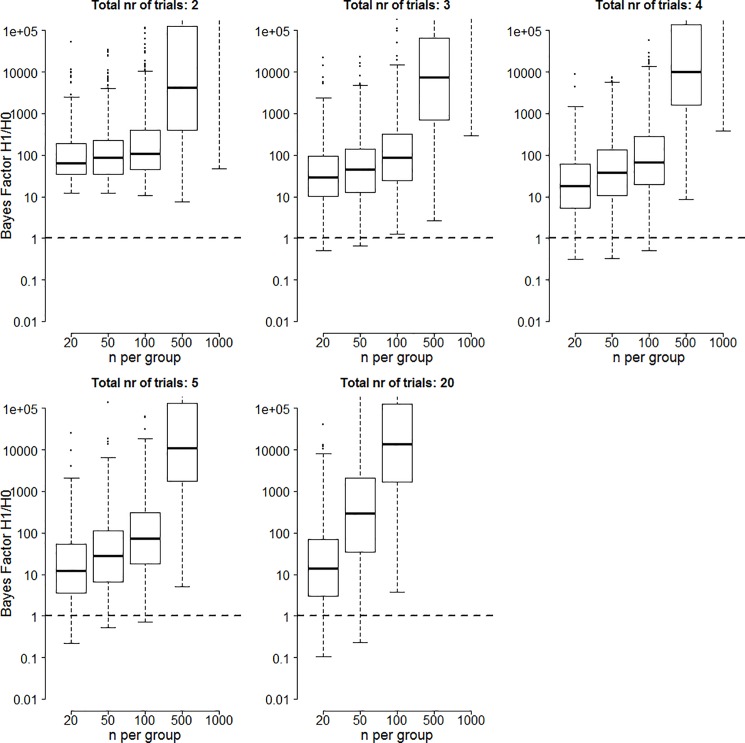
Bayes factors in favor of the alternative hypothesis for two significant trials when the true effect size is 0.2. Boxes contain Bayes factors for 50% of the simulations with tails extending to Bayes factors for 100% of the simulations excluding outliers. Circles indicate outliers and are those values more than 1.5*IQR removed from the boxes. Note that for large numbers of participants, Bayes factors increase exponentially and only the tail of the boxes is visible.

The Bayes factor results of the small effect size simulations are shown in Fig 1. In all panels, the y-axis plots the Bayes factor in favor of the alternative hypothesis on a log scale. Different panels indicate different number of trials, and different columns indicate a different number of participants. The box-plots contain the middle 50% of simulation results, with the tails extending to 100% of the simulation results excluding outliers. The horizontal dashed line represents the case where evidence equally favors the alternative and the null hypothesis, results above the line favor the alternative hypothesis, and results below the line favor the null hypothesis. Note that the cells for 5 trials and 1000 participants, 20 trials and 500 participants, and 20 trials and 1000 participants are empty. The reason for this is that these conditions do not realistically occur for this effect size.

The results show that there is substantial variability in the evidential strength both across different types of simulations (as reflected by the different heights of the boxes) and within different types of simulations (as reflected by the size of the boxes and the extent of the tails). Summarizing the main trends, the evidential strength for medications that achieve two trials with statistically significant results is lower if more trials were conducted (boxes get lower for higher number of trials), the evidential strength is higher if more participants were tested in the placebo and experimental groups (boxes get higher to the right of each panel), and we see an interaction between number of trials and number of participants: increasing the number of participants has a stronger effect for a larger number of trials.

**Fig 2:**
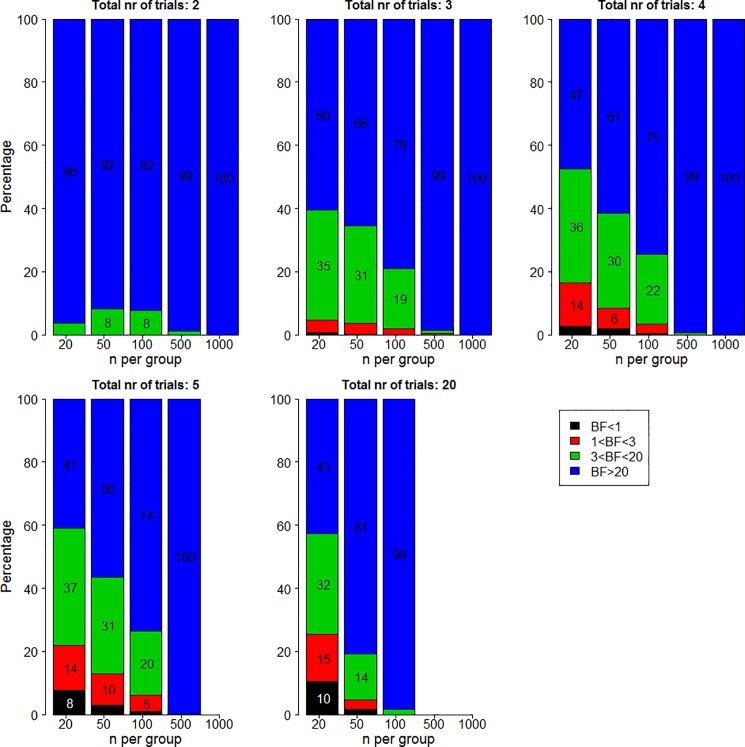
Percentage of Bayes factors in favor of the alternative hypothesis lower than 1 (black), between 1 and 3 (red), between 3 and 20 (green), and higher than 20 (blue) for two significant trials when the true effect size is 0.2.

How often would it happen that medications that achieve two trials with statistically significant results actually have the overall evidence point in favor of the null hypothesis? The answer can be found in Fig 2. In all panels, the y-axis plots the percentage of 500 simulations for which the Bayes factor is lower than a certain cut-off value. Different panels indicate different number of trials, different columns indicate different number of participants, and different colors indicate different cut-off values.

As shown in the bottom left of the bottom middle panel, when the number of trials is 20 and the number of participants per group is 20, in 10.4% of all simulations the evidence is actually in favor of the null (i.e., the black part of the bar-plot). A BF that does not exceed 3 in favor of the alternative hypothesis over the null hypothesis is quite a common occurrence for scenarios with relatively few participants or a relatively large number of trials (i.e., the black and the red part of the bar-plot). For instance, with 2 out of 5 significant trials and 20 participants per group, our strength of evidence in favor of the alternative hypothesis is lower than 3 in 22% of all simulations. Finally, examining the combined black, red, and green parts of the bar-plot shows that a BF not exceeding 20 is very common. Depending on the number of participants and the number of trials, the percentage of simulations that have strength of evidence in favor of the alternative hypothesis that is lower than 20 can be over half of all simulations.

**Fig 3:**
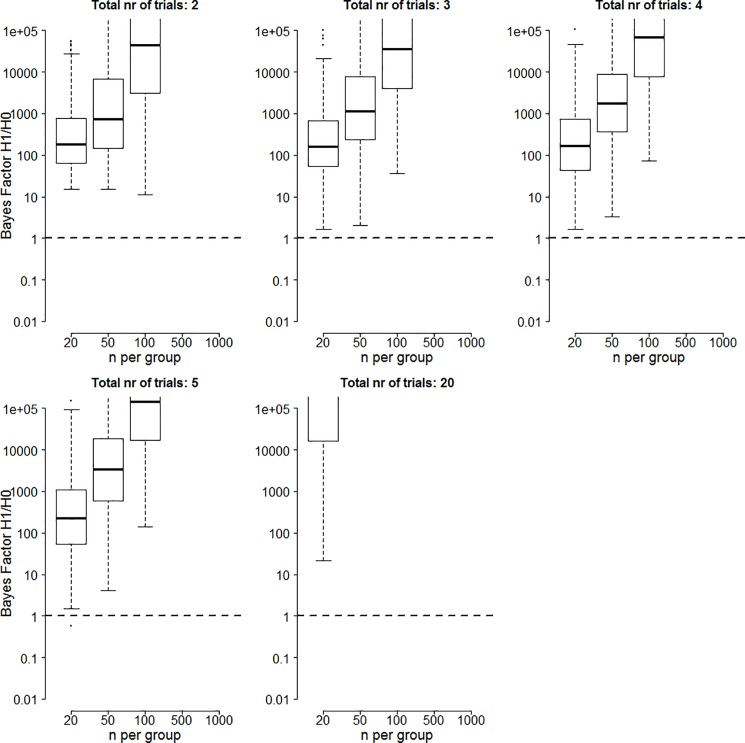
Bayes factors in favor of the alternative hypothesis for two significant trials when the true effect size is 0.5. Boxes contain Bayes factors for 50% of the simulations with tails extending to Bayes factors for 100% of the simulations excluding outliers. Circles indicate outliers and are those values more than 1.5*IQR removed from the boxes.

The median Bayes factor results of the medium effect size simulations are shown in Fig 3. The layout is similar to that of Fig 1. Note that all cells for n = 500 and n = 1000, the cell for 20 trials and 50 participants, and the cell for 20 trials and 100 participants are empty.

Comparing these results to those obtained in Fig 1 shows that median Bayes factors are higher for a higher effect size. Increasing the number of participants also has a stronger influence on the Bayes factor when the true effect size is larger.

**Fig 4:**
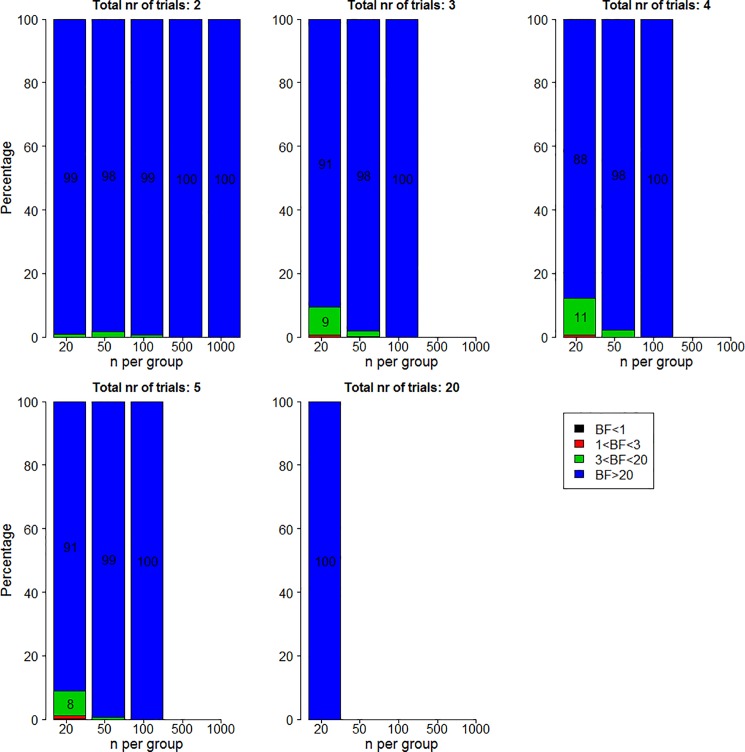
Percentage of Bayes factors in favor of the alternative hypothesis lower than 1 (black), between 1 and 3 (red), between 3 and 20 (green), and higher than 20 (blue) for two significant trials when the true effect size is 0.5.

The percentage of 500 simulations for which the Bayes factor is lower than a certain cut-off value for median effect size is displayed in Fig 4. The layout is similar to that of Fig 2. Examining the results shows that if the true effect size is 0.5, Bayes factor are rarely lower than 3 in favor of the alternative and are often larger than 20.

**Fig 5:**
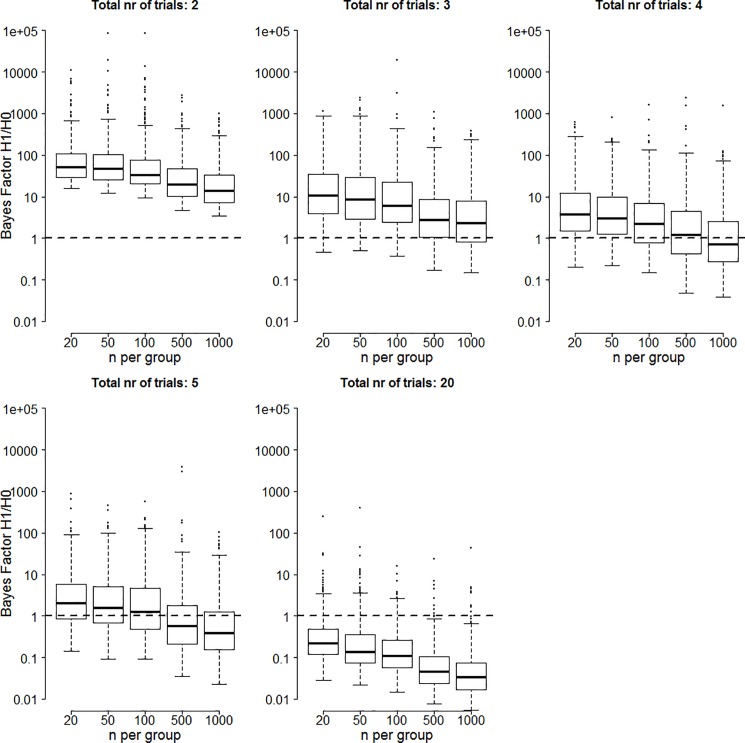
Bayes factors in favor of the alternative hypothesis for two significant trials when the true effect size is 0. Boxes contain Bayes factors for 50% of the simulations with tails extending to Bayes factors for 100% of the simulations excluding outliers. Circles indicate outliers and are those values more than 1.5*IQR removed from the boxes.

The median Bayes factor results of the zero effect size simulations are shown in Fig 5. The layout is similar to that of Figs 1 and 3.

As shown, when the true effect size is zero, the Bayes factor becomes much lower than for scenarios where the true effect size is non-zero. In most simulation cells, the median Bayes factor is lower than 10 and in many cases the Bayes factor actually favors the null hypothesis. This is particularly true when the number of trials is larger and the number of participants is larger.

**Fig 6:**
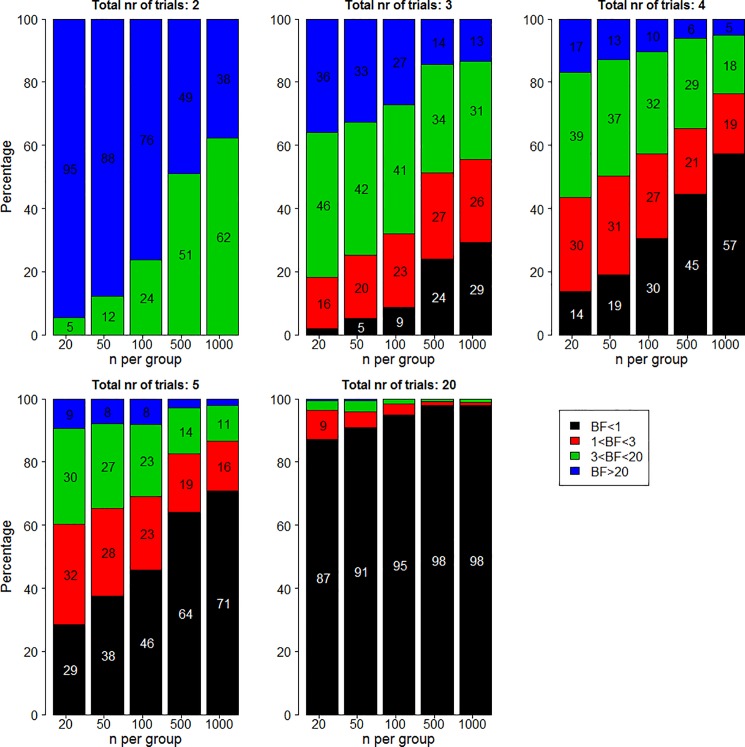
Percentage of Bayes factors in favor of the alternative hypothesis lower than 1 (left panel), lower than 3 (middle panel), and lower than 20 (right panel) for two significant trials when the true effect size is 0.

The percentage of 500 simulations for which the Bayes factor is lower than a certain cut-off value for zero effect size is displayed in Fig 6. The layout is similar to that of Figs 2 and 4, but note that in this case, black parts represent the outcomes that align most closely with reality and blue parts represent the outcomes that align least closely with reality.

Examining the results shows that if the true effect size is zero, Bayes factor are usually in favor of the null hypothesis if the number of trials is large. When the true effect is zero, Bayes factors are rarely larger than 20, except in cases where 2 out of 2 trials were significant, but this should be a rare occurrence if the true effect size is zero. In additional simulations, we verified that our results hold in case of variable underlying effect size distributions and in case of unequal variance in the experimental group compared to the control group (see online supplement).

When taken together, the results provide striking evidence of the large variety in strength of evidence that occurs as a result of a strict endorsement criterion of exactly two *p*-values lower than .05. When using this criterion, the strength of evidence is generally larger when the underlying effect size is larger and when the number of participants is higher. Medicines should only be endorsed if the evidence in favor of its efficacy is strong and consistent, but the reality provides a stark contrast to that ideal.

## Discussion

In this study, we simulated clinical trial data comparing an experimental group to a placebo. Simulations differed on the underlying true effect size, the number of clinical trials, and the number of participants in each trial. The simulations all had one thing in common: exactly two of the conducted trials were statistically significant with a two-tailed *p*-value lower than .05 and an effect in the expected direction (favoring the experimental intervention). For all simulations, we assessed the strength of evidence in favor of the alternative, the experimental group outperforms the placebo, by means of a Bayes factor using a default prior.

The result of our simulations is simple yet compelling: a criterion of endorsement of two *p*-values lower than .05 leads to a large variety in strength of evidence in favor of a medicine’s efficacy. In a non-trivial proportion of cases, this criterion even leads to endorsement when statistical evidence actually favors the null hypothesis. Bayes factors that favor the null hypothesis for two *p*-values lower than .05 occur when the true effect size is zero, when the number of trials is large, and when the number of participants in both groups is low.

What are we to conclude from this? First and foremost, it is likely that a criterion asking for two statistically significant trials would often lead to correct endorsement of new medications. It is difficult to estimate the true proportion of incorrectly endorsed medicine, because the underlying true effect size cannot be known in advance. However, empirical evidence across many medical interventions suggests that most effects are small or modest [19]. Our results show that even for a true effect size of .5, medicines sometimes get endorsed based on very unconvincing evidence. This result is a direct consequence of the *p*-value’s propensity to over-reject the null hypothesis [6].

Fortunately, a straightforward solution exists: quantifying evidence using the Bayes factor. Such a change in protocol for statistical inference is not unprecedented. Use of Bayesian statistics in clinical trials design and analysis has been used for a number of years by FDA in some domains (e.g., medical device clinical trials; [22–24]). Use of Bayesian methods for statistical inference has been widely recommended for a long time [25,26], but has sparsely been endorsed [2]. Computational difficulties are no longer an excuse for the underuse of Bayesian methods. With the advent in computational power in recent decades came the possibility of computer-driven sampling routines to approximate posterior distributions necessary to calculate Bayes factors in the form of so-called Markov chain Monte Carlo sampling (MCMC)[27,28].

Another important problem that was not solved until recently was the absence of easy-to-handle statistical software with an intuitive interface. This meant that the application of the Bayesian hypothesis test was a tool that could only be used by statistical experts. The recent development of online Bayes factor calculator tools [20] (tools available at http://pcl.missouri.edu/bayesfactor) and the statistical freeware program JASP [29] has greatly enhanced the accessibility of Bayesian statistical inference.

It is important to stress that the results of our simulations make no assumptions as to how the data were obtained. Our simulations make no commitment about the nature of the data, whether it was obtained with honest intentions, through cherry-picking, or through *p*-hacking. Our results are in agreement with some empirical data on placebo-controlled trials submitted to the FDA. For example, Monden et al. examined 58 trials of second-generation antidepressants for generalized anxiety [30]. The BF estimated for these trials varied widely from 0.07 to 131000. Among the 59 doses that were felt by the FDA to have substantial evidence for efficacy, only 26 had a BF of at least 20.

Some limitations of our study should be discussed. First, the “exactly 2 significant trials” rule that we simulated may not capture fully the way that FDA or other regulatory agencies operate, even without explicit consideration of Bayesian methods. The FDA’s position to require “…at least two adequate and well-controlled studies, each convincing on its own, to establish effectiveness” is not the same as “exactly 2 significant trials”. Our demonstration provides an indication of the strength of evidence one were to obtain when this policy is employed with exactly two significant trials. Furthermore, the approval process includes consideration of multiple aspects of efficacy and safety and it also entails a qualitative assessment of the adequacy of design, conduct and analysis of the trial and of the relevance of the outcomes used. Therefore, our simulations should not be seen as exactly mapping the regulatory process, but rather as exploring the consequences of using a rule that is based on statistical significance alone. Safety assessment in particular has a stronger track record of use of Bayesian analysis [31].

Second, there can be differences of opinion on how strong the evidence should be before a medication is approved. A BF of 3 typically is considered to be very weak and worth mere mentioning, while even a BF of 20 may not be considered conclusive at times [17].

Third, we used two different non-null effect sizes, but the magnitude of the effect that is considered to be sufficiently good to lead to approval may vary on a case-by-case basis. E.g. the type of outcome, the availability of other drugs, the safety profile of the tested medication, and other factors may also be involved in the decision-making.

Fourth, we have focused on evaluating superiority trials, but for some drugs decisions may be made based on non-inferiority designs. Non-inferiority trials are a minority: A survey identified 209 non-inferiority or equivalence trials published in 2009 [32]. Appropriate Bayesian considerations apply also for non-inferiority trials [33,34].

Fifth, we used a standard Bayesian framework for all analyses, so as to standardize the inferences derived. In real practice, some further diversification can exist based on additional prior evidence. For our simulations, we assumed non-informative priors.

Allowing for these caveats, our study offers through simulations yet another demonstration of the unfortunate effect of *p*-values on statistical inferences. More routine consideration of BF in regulatory assessments and clinical decision-making would be a step forward for the adoption of medications in clinical practice.

## Supporting information

S1 Fig Bayes factors in favor of the alternative hypothesis for two significant trials when the true effect size is variable. Boxes contain Bayes factors for 50% of the simulations with tails extending to Bayes factors for 100% of the simulations. Note that for large numbers of participants, Bayes factors increase exponentially and only the tail of the boxes is visible.
(TIF)

S2 Fig Proportion of Bayes factors in favor of the alternative hypothesis lower than 1 (black), between 1 and 3 (red), between 3 and 20 (green), and higher than 20 (blue) for two significant trials when the true effect size is variable. (TIF)

S3 Fig Bayes factors in favor of the alternative hypothesis for two significant trials when the true effect size is 0.2 and the standard deviation is not equal between groups. Boxes contain Bayes factors for 50% of the simulations with tails extending to Bayes factors for 100% of the simulations.
(TIF)

S4 Fig Proportion of Bayes factors in favor of the alternative hypothesis lower than 1 (black), between 1 and 3 (red), between 3 and 20 (green), and higher than 20 (blue) for two significant trials when the true effect size is 0.2 and the standard deviation is not equal between groups. (TIF)

S5 Fig Bayes factors in favor of the alternative hypothesis for two significant trials when the true effect size is 0.5 and the standard deviation is not equal between groups. Boxes contain Bayes factors for 50% of the simulations with tails extending to Bayes factors for 100% of the simulations.
(TIF)

S6 Fig Proportion of Bayes factors in favor of the alternative hypothesis lower than 1 (black), between 1 and 3 (red), between 3 and 20 (green), and higher than 20 (blue) for two significant trials when the true effect size is 0.5 and the standard deviation is not equal between groups. (TIF)

S7 Fig Bayes factors in favor of the alternative hypothesis for two significant trials when the true effect size is 0 and the standard deviation is not equal between groups. Boxes contain Bayes factors for 50% of the simulations with tails extending to Bayes factors for 100% of the simulations.
(TIF)

S8 Fig Proportion of Bayes factors in favor of the alternative hypothesis lower than 1 (black), between 1 and 3 (red), between 3 and 20 (green), and higher than 20 (blue) for two significant trials when the true effect size is 0 and the standard deviation is not equal between groups. (TIF)

S9 Fig Bayes factors in favor of the alternative hypothesis for two significant trials when the true effect size is variable and the standard deviation is not equal between groups. Boxes contain Bayes factors for 50% of the simulations with tails extending to Bayes factors for 100% of the simulations.
(TIF)

S10 Fig Proportion of Bayes factors in favor of the alternative hypothesis lower than 1 (black), between 1 and 3 (red), between 3 and 20 (green), and higher than 20 (blue) for two significant trials when the true effect size is variable and the standard deviation is not equal between groups. (TIF)

S1 File van Ravenzwaaij & Ioannidis, 2017, Supplementary. (PDF)

S2 File FDAweb.r. (R)

## References

[1] Katz R. FDA: Evidentiary standards for drug development and approval. NeuroRx. 2004;1: 307–316. 10.1602/neurorx.1.3.307 15717032 PMC534930

[2] Chavalarias D, Wallach JD, Li AHT, Ioannidis JPA. Evolution of reporting p values in the biomedical literature, 1990–2015. J Am Med Assoc. 2016;315: 1141–1148. 10.1001/jama.2016.1952 26978209

[3] Goodman SN. P values, hypothesis tests, and likelihood: implications for epidemiology of a neglected historical debate. Am J Epidemiol. 1993;137: 485–496. 8465801 10.1093/oxfordjournals.aje.a116700

[4] Goodman SN. Toward evidence-based medical statistics. 1: The P value fallacy. Ann Intern Med. 1999;130: 995–1004. 10383371 10.7326/0003-4819-130-12-199906150-00008

[5] Greenland S, Senn SJ, Rothman KJ, Carlin JB, Poole C, Goodman SN, Altman DG. Statistical tests, p values, confidence intervals, and power: a guide to misinterpretations. Eur J Epidemiol. 2016;31: 337–350. 10.1007/s10654-016-0149-3 27209009 PMC4877414

[6] Ioannidis JPA. Why most published research findings are false. PLOS Med. 2005;2: e124 10.1371/journal.pmed.0020124 16060722 PMC1182327

[7] Wasserstein RL, Lazar NA. The ASA’s statement on p-values: context, process, and purpose. Am Stat. 2016;70: 129–133.

[8] Gigerenzer G. Mindless statistics. J Socio Econ. 2004;33: 587–606.

[9] Hoekstra R, Morey RD, Rouder JN, Wagenmakers E-J. Robust misinterpretation of confidence intervals. Psychon Bull Rev. 2014;21: 1157–1164. 10.3758/s13423-013-0572-3 24420726

[10] Wagenmakers E-J. A practical solution to the pervasive problems of p values. Psychon Bull Rev. 2007;14: 779–804. 18087943 10.3758/bf03194105

[11] Mueller PS, Montori VM, Bassler D, Koenig BA, Guyatt GH. Ethical issues in stopping randomized trials early because of apparent benefit. Ann Intern Med. 2007;146: 878–881. 17577007 10.7326/0003-4819-146-12-200706190-00009

[12] Ioannidis JPA. Khoury MJ. Improving validation practices in “Omics” Research. Science. 2011;334: 1230–1232. 10.1126/science.1211811 22144616 PMC5624327

[13] GRADE working group. GRADE: what is “quality of evidence” and why is it important to clinicians? BMJ. 2008;336: 995–998. 10.1136/bmj.39490.551019.BE 18456631 PMC2364804

[14] Food and Drug Administration. Guidance for industry: providing clinical evidence of effectiveness for human drug and biological products. Maryland: United States Food and Drug Administration;1998.

[15] Jeffreys H. Theory of probability. 3rd ed. Oxford, UK: Oxford University Press; 1998.

[16] Goodman SN. Toward evidence-based medical statistics. 2: the Bayes factor. Ann Intern Med. 1999;130: 1005–1013. 10383350 10.7326/0003-4819-130-12-199906150-00019

[17] Kass RE, Raftery AE. Bayes factors. J Am Stat Assoc. 1995;90: 773–795.

[18] Sullivan GM, Feinn R. Using Effect Size- or Why the P Value Is Not Enough. J Grad Med Educ. 2012;4: 279–282. 10.4300/JGME-D-12-00156.1 23997866 PMC3444174

[19] Pereira TV, Horwitz RI, Ioannidis JPA. Empirical evaluation of very large treatment effects of medical interventions. J Am Med Assoc. 2012;308: 1676–1684. 10.1001/jama.2012.13444 23093165

[20] Rouder JN, Speckman PL, Sun D, Morey RD, Iverson G. Bayesian t tests for accepting and rejecting the null hypothesis. Psychon Bull Rev. 2009;16: 225–37. 10.3758/PBR.16.2.225 19293088

[21] O’Hagan A., Forster J. Kendall’s Advanced Theory of Statistics, Vol. 2B: Bayesian Inference (2nd edn.). London: Arnold;2004.

[22] Pennello G, Thompson L. Experience with reviewing Bayesian medical device trials. J Biopharm Stat. 2008;18: 81–115. 10.1080/10543400701668274 18161543

[23] Campbell G. Bayesian statistics in medical devices: innovation sparked by the FDA. J Biopharm Stat. 2011;21: 871–887. 10.1080/10543406.2011.589638 21830920

[24] US Food and Drug Administration. Drugs@FDA: FDA approved drug products [internet]. United States Food and Drug Administration; 2016 [cited 2016 Jun 2]. Available from http://www.accessdata.fda.gov/scripts/cder/drugsatfda/index.cfm

[25] Hobbs BP, Carlin BP. Practical Bayesian design and analysis for drug and device clinical trials. J Biopharm Stat. 2008;18: 54–80. 10.1080/10543400701668266 18161542

[26] Zaslavsky BG. Bayesian Hypothesis Testing in Two-Arm Trials with Dichotomous Outcomes. Biometrics. 2013;69: 157–163. 10.1111/j.1541-0420.2012.01806.x 23002906

[27] Gamerman D, Lopes HF. Markov chain Monte Carlo: Stochastic simulation for Bayesian inference. 2nd Ed. Florida: Chapman Hall; 2006.

[28] van Ravenzwaaij D, Cassey P, Brown SD. A Simple Introduction to Markov Chain Monte-Carlo. Psychon Bull Rev. 2016. 10.3758/s13423-016-1015-8 PMC5862921 26968853

[29] JASP [computer program]. Version 0.7.5.6. The JASP Team; 2016. https://jasp-stats.org/.

[30] Monden R, de Vos S, Morey R, Wagenmakers E-J, de Jonge P, Roest AM. Toward evidence-based medical statistics: a Bayesian analysis of double-blind placebo-controlled antidepressant trials in the treatment of anxiety disorders. Int J Methods Psychiatr Res. 2016. 10.1002/mpr.1507 PMC6860243 27219132

[31] Scott JA, Hand AL, Sian LS. BayesWeb: a user-friendly platform for exploratory Bayesian analysis of safety signals from small clinical trials. J Biopharm Stat. 2011;21: 1030–1041. 10.1080/10543406.2011.590924 21830929

[32] Schiller P, Burchardi N, Niestroj M, Kieser M. Quality of reporting of clinical non-inferiority and equivalence randomized trials-update and extension. Trials. 2012;13: 214 10.1186/1745-6215-13-214 23157733 PMC3554513

[33] Gamalo-Siebers M, Gao A, Lakshminarayanan M, Liu G, Natanegara F, Railkar R, Schmidli H, Song G. Bayesian methods for the design and analysis of noninferiority trials. J Biopharm Stat. 2016;26: 1–19. 26247350 10.1080/10543406.2015.1074920

[34] Gamalo MA, Wu R, Tiwari RC. Bayesian approach to non-inferiority trials for normal means. Stat Methods Med Res. 2016;25: 221–240. 10.1177/0962280212448723 22619277

