# Supplementary figures and images for: A simulation study of the strength of evidence in the recommendation of medications based on two trials with statistically significant results
Source: PLoS One. 2017 Mar 8;12(3):e0173184. doi: 10.1371/journal.pone.0173184 (PMC5342224; doi:10.1371/journal.pone.0173184)

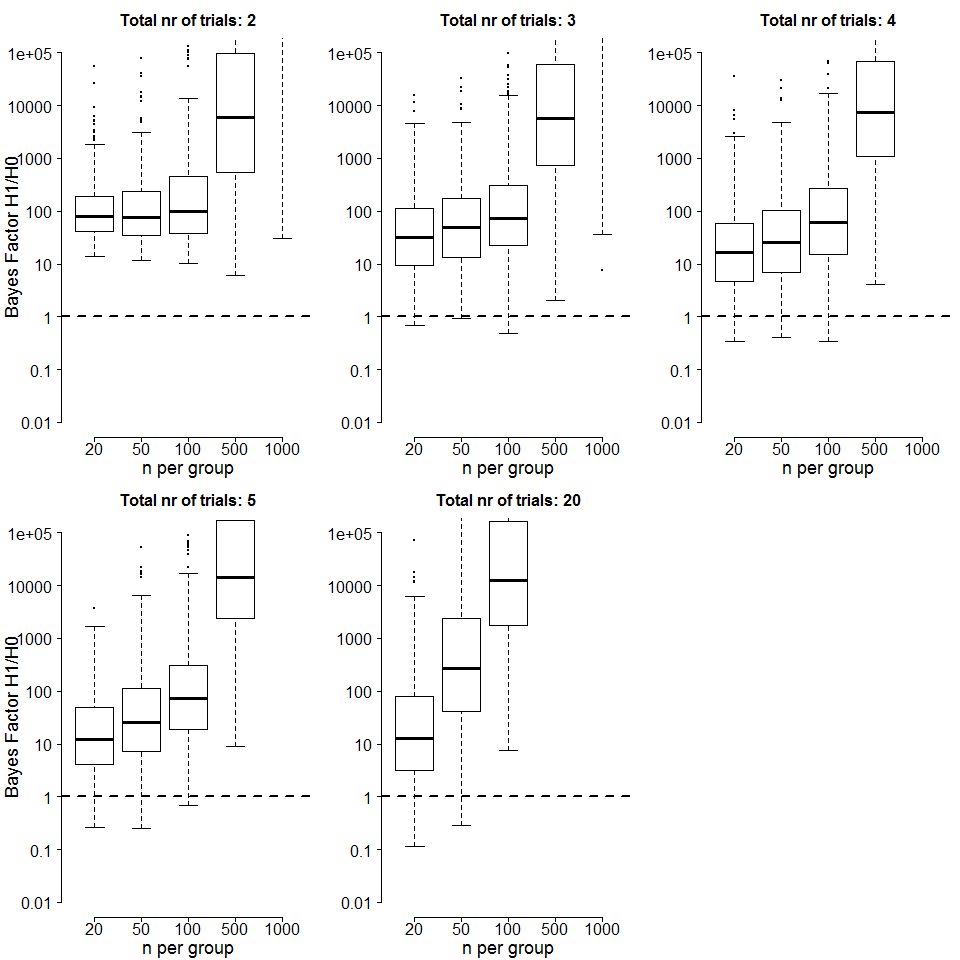

Supplement: S1 Fig — Boxes contain Bayes factors for 50% of the simulations with tails extending to Bayes factors for 100% of the simulations. Note that for large numbers of participants, Bayes factors increase exponentially and only the tail of the boxes is visible. (TIF) [file pone.0173184.s001.tif]

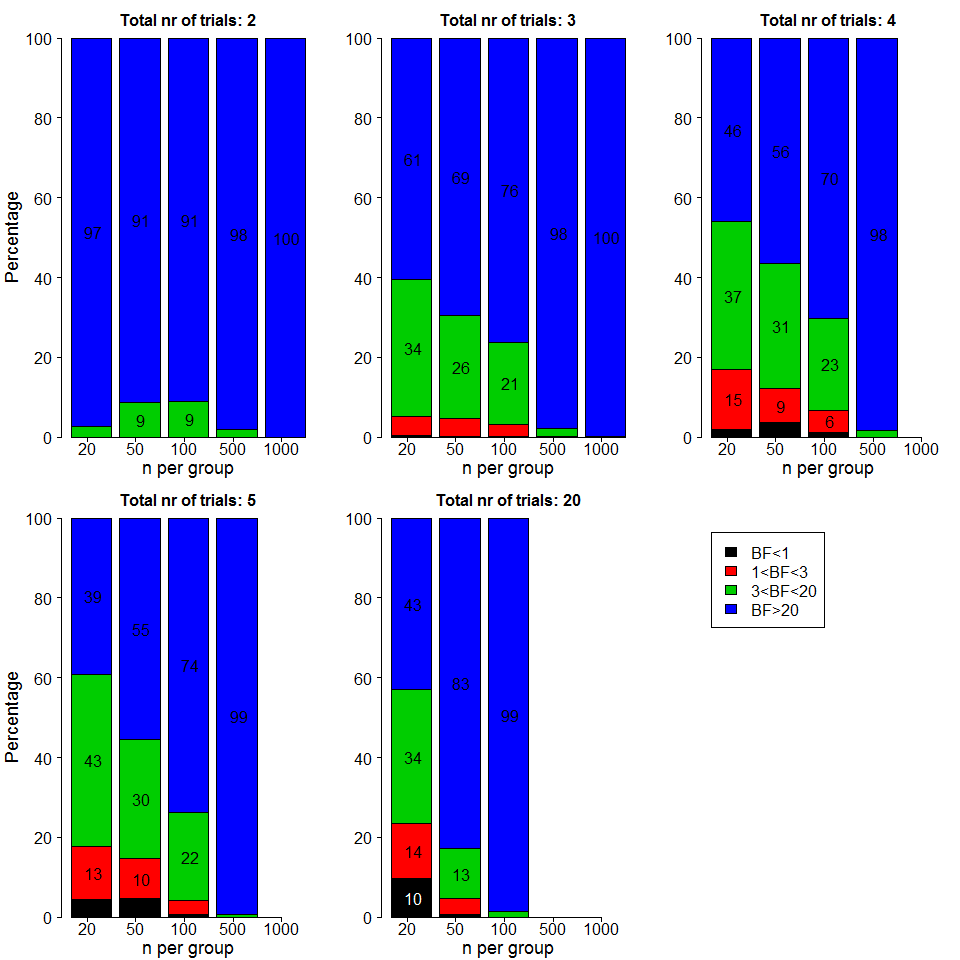

Supplement: S2 Fig — (TIF) [file pone.0173184.s002.tif]

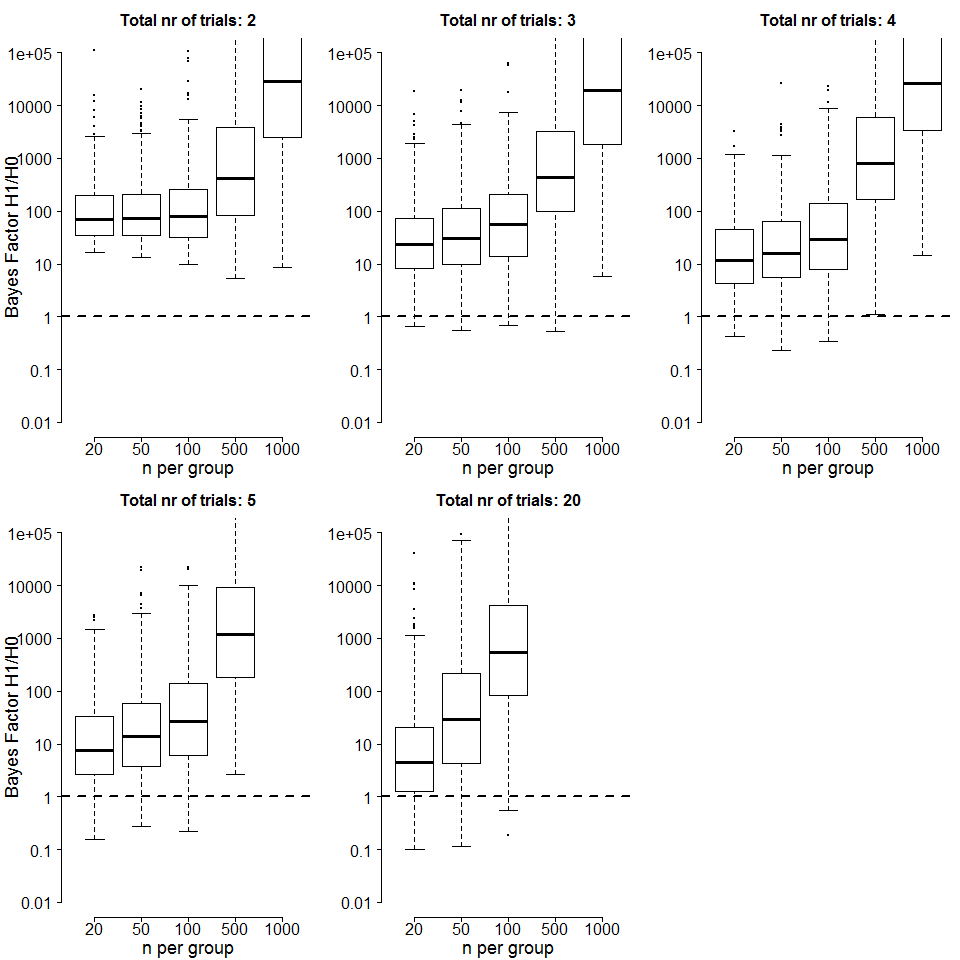

Supplement: S3 Fig — Boxes contain Bayes factors for 50% of the simulations with tails extending to Bayes factors for 100% of the simulations. (TIF) [file pone.0173184.s003.tif]

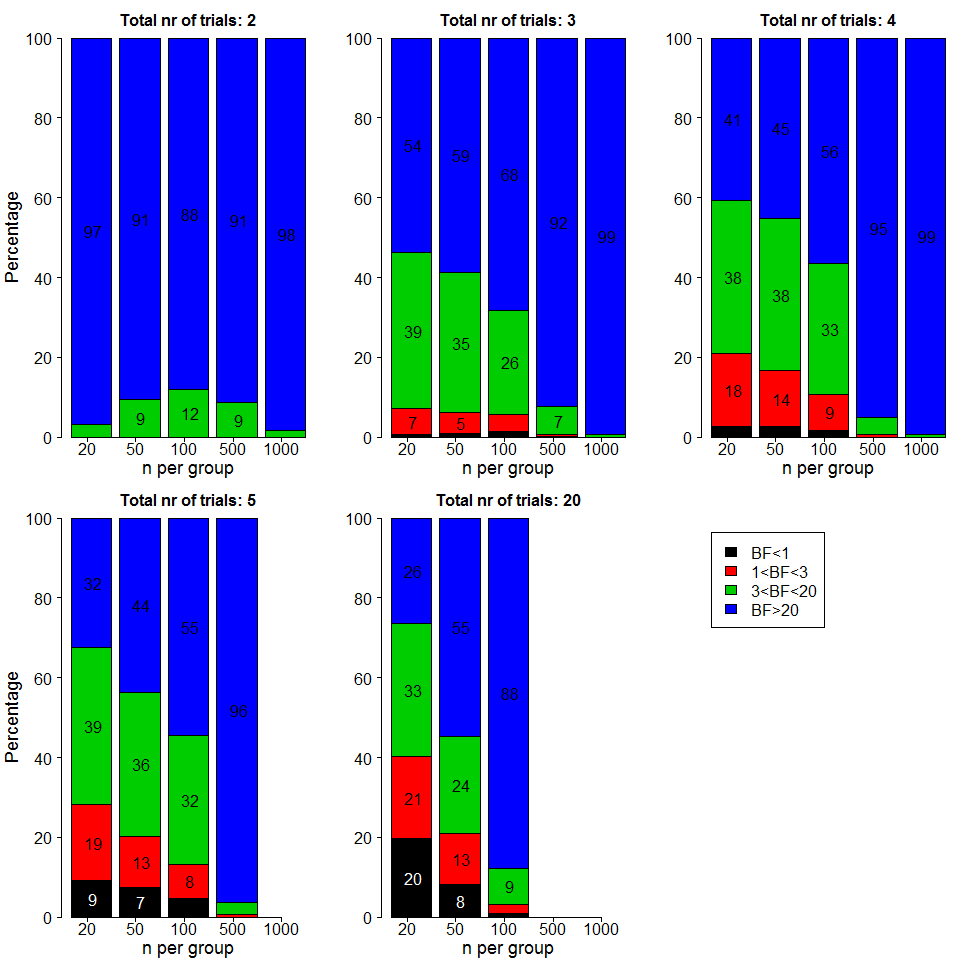

Supplement: S4 Fig — (TIF) [file pone.0173184.s004.tif]

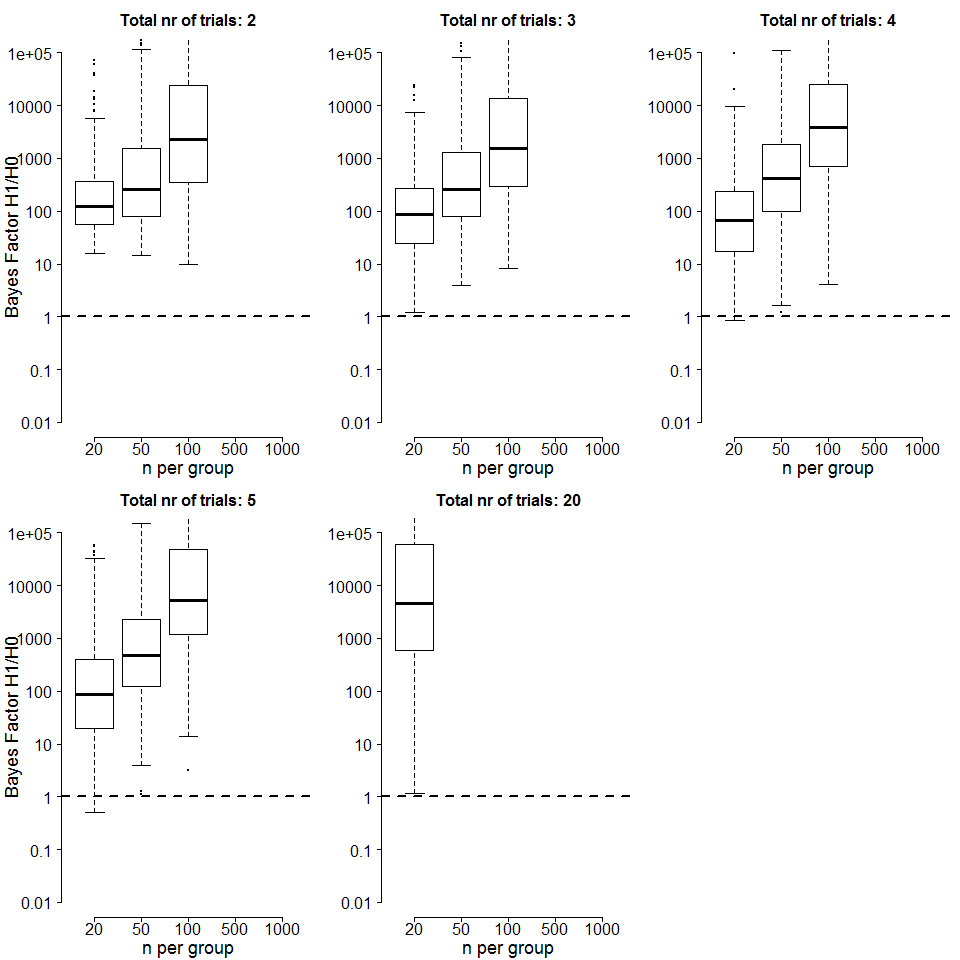

Supplement: S5 Fig — Boxes contain Bayes factors for 50% of the simulations with tails extending to Bayes factors for 100% of the simulations. (TIF) [file pone.0173184.s005.tif]

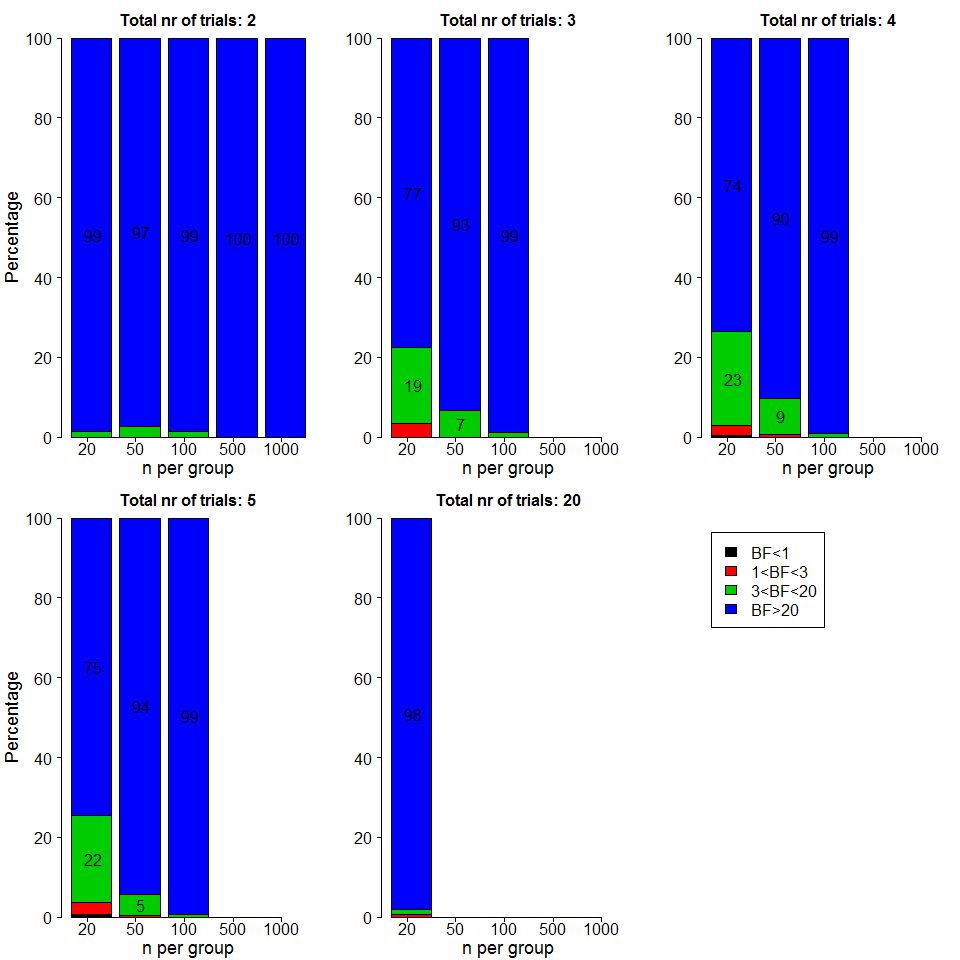

Supplement: S6 Fig — (TIF) [file pone.0173184.s006.tif]

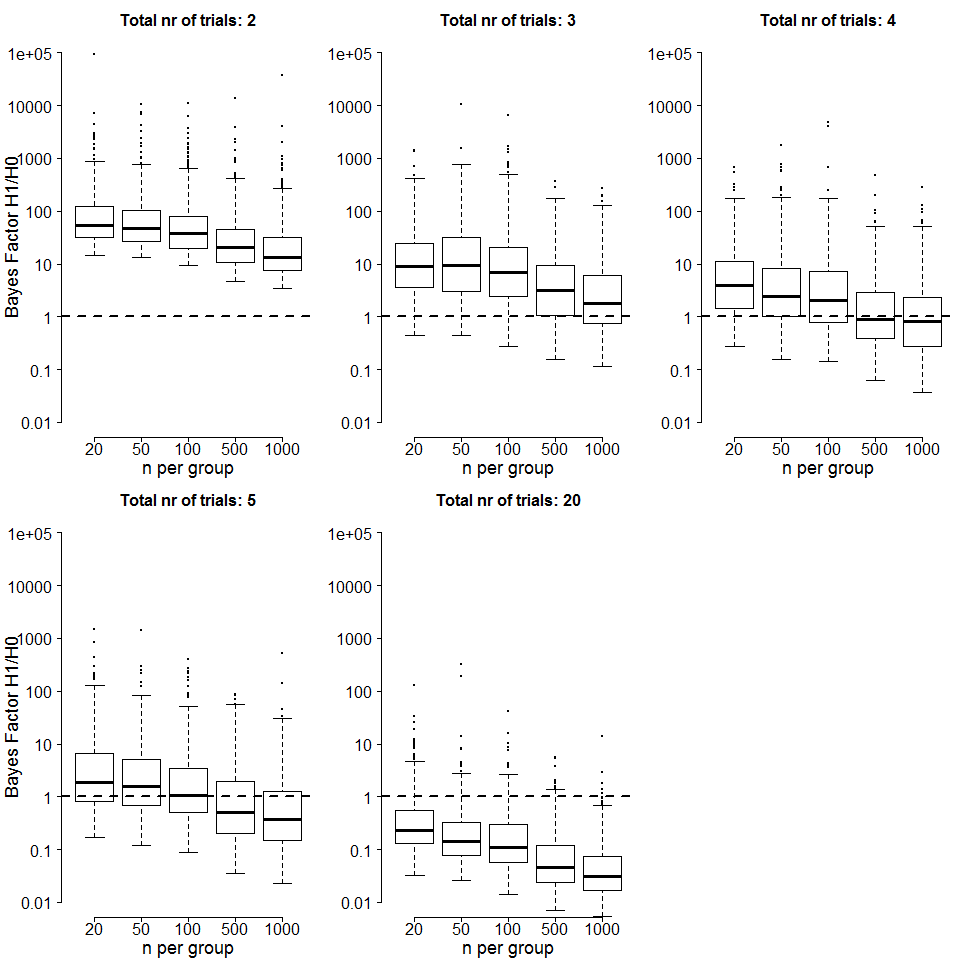

Supplement: S7 Fig — Boxes contain Bayes factors for 50% of the simulations with tails extending to Bayes factors for 100% of the simulations. (TIF) [file pone.0173184.s007.tif]

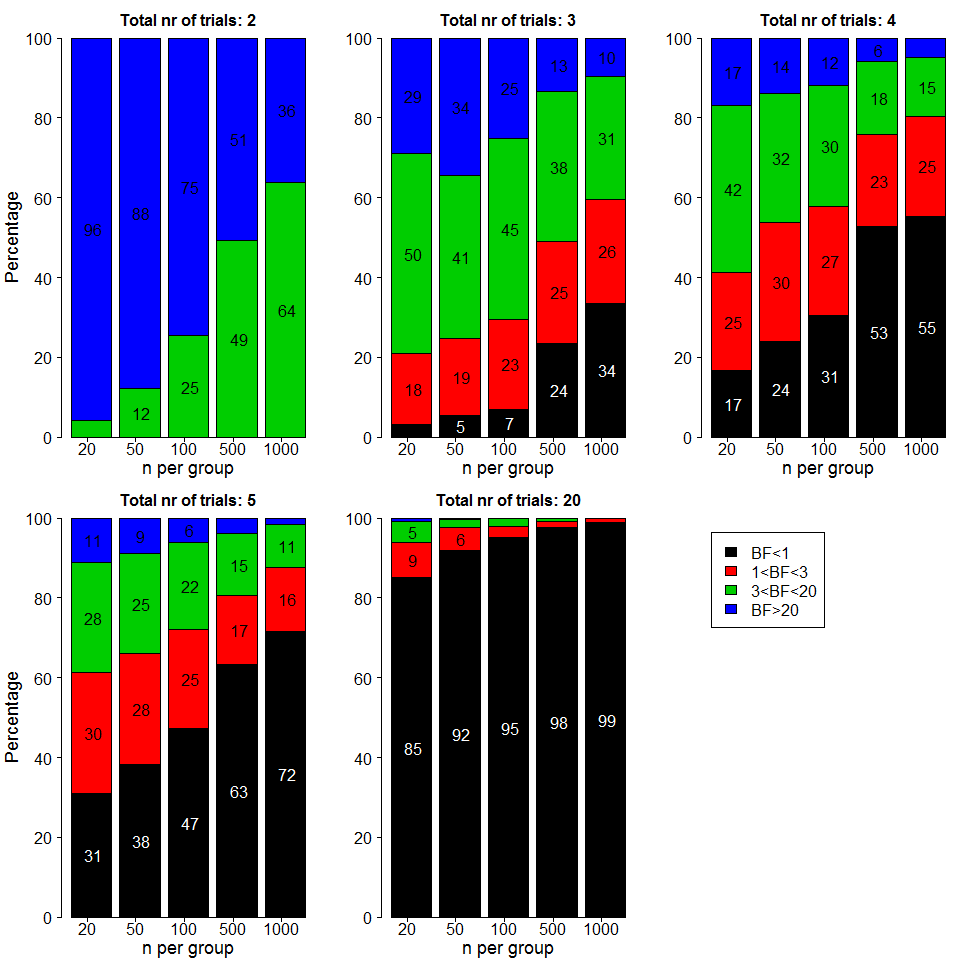

Supplement: S8 Fig — (TIF) [file pone.0173184.s008.tif]

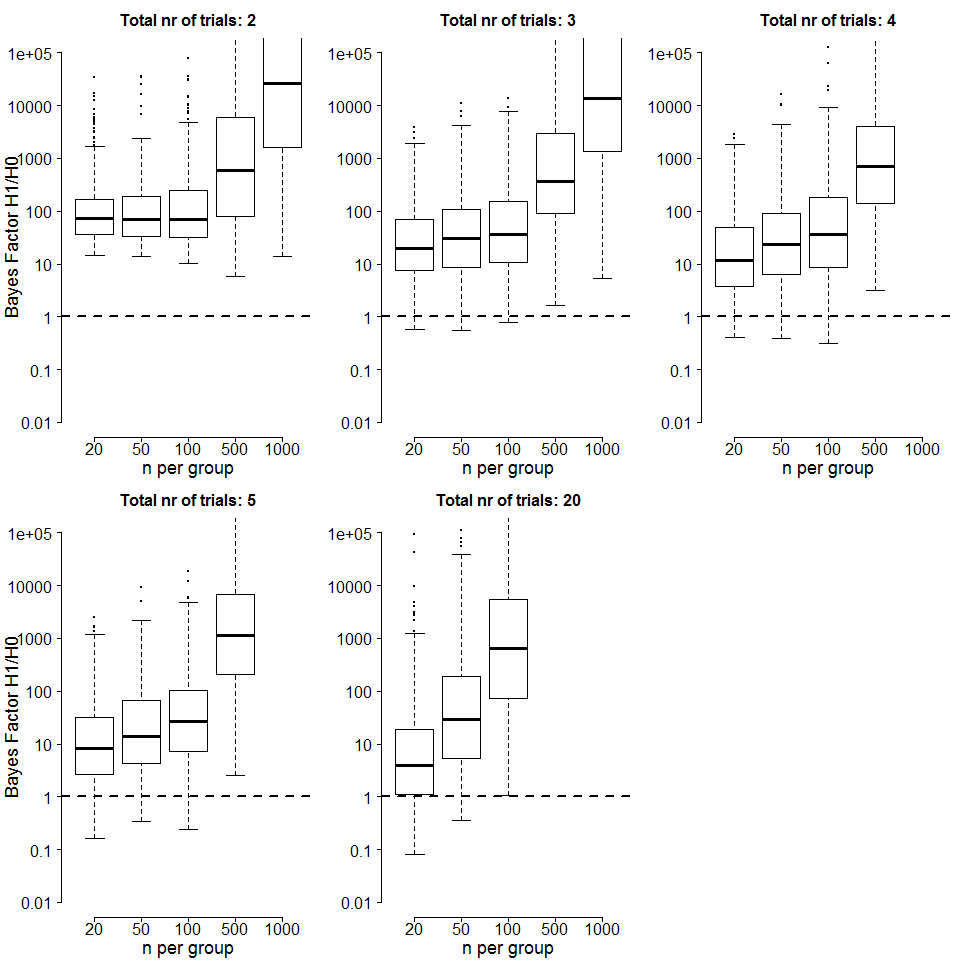

Supplement: S9 Fig — Boxes contain Bayes factors for 50% of the simulations with tails extending to Bayes factors for 100% of the simulations. (TIF) [file pone.0173184.s009.tif]

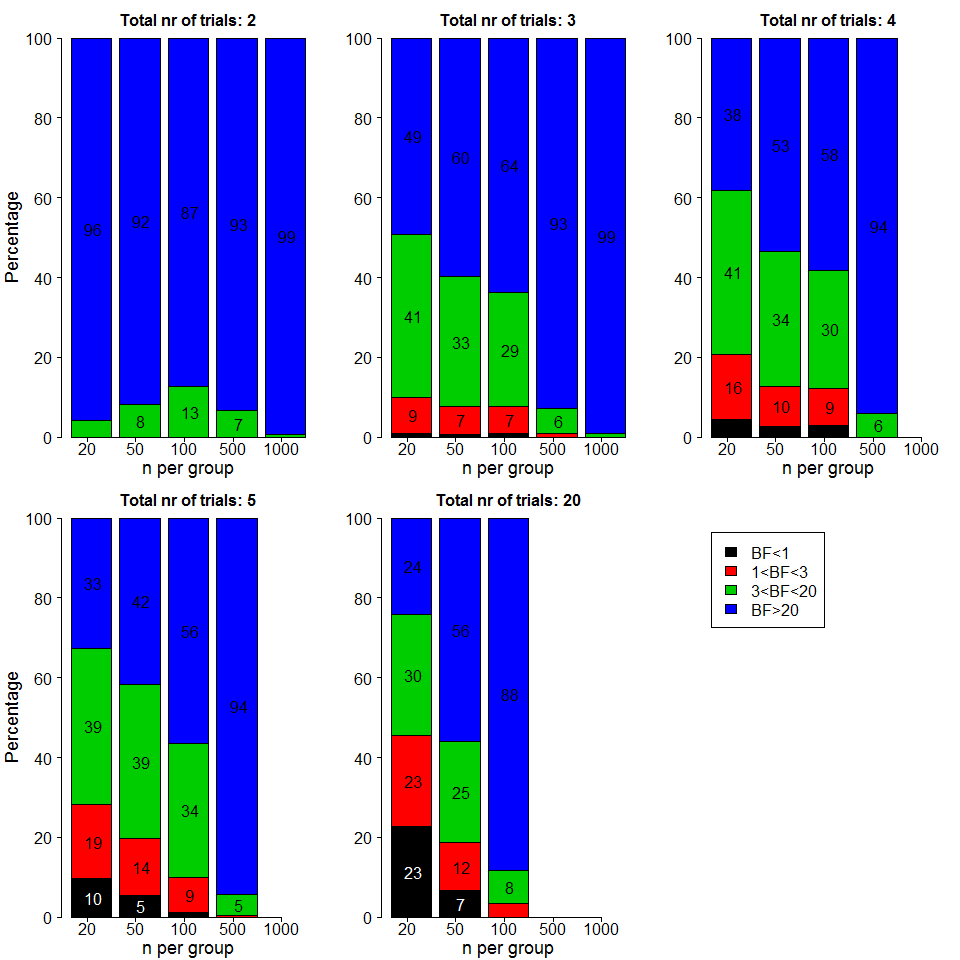

Supplement: S10 Fig — (TIF) [file pone.0173184.s010.tif]
