# Supplementary material for: A simulation study of the strength of evidence in the recommendation of medications based on two trials with statistically significant results
Source: PLoS One. 2017 Mar 8;12(3):e0173184. doi: 10.1371/journal.pone.0173184 (PMC5342224; doi:10.1371/journal.pone.0173184)
Supplement: S1 File — (PDF) [file pone.0173184.s011.pdf]

SUPPLEMENTARY MATERIAL

A Simulation Study of the Strength of Evidence in the Endorsement of Medications Based on Two Trials with Statistically Significant Results

Don van Ravenzwaaij[1] and John P. A. Ioannidis[2]
[1]Department of Psychology, University of Groningen, the Netherlands [2]Departments of Medicine, of Health Research and Policy, and of Statistics and Meta-Research Innovation Center at Stanford (METRICS), Stanford University, USA

Correspondence concerning this article should be addressed to:
Don van Ravenzwaaij
University of Groningen, Department of Psychology
Grote Kruisstraat 2/1, Heymans Building, room 169
9712 TS Groningen, The Netherlands
Ph: (+31) 50 363 7021
E–mail should be sent to d.van.ravenzwaaij@rug.nl.

This document contains supplementary material to "A Simulation Study of the Strength of Evidence in the Endorsement of Medications Based on Two Trials with Statistically Significant Results" [1]. Included are extra simulations that manipulate two things that were not examined in the main manuscript: (1) individual differences in the underlying effect size distribution and (2) different variance in the experimental group compared to the control group. For details about the general simulation set-up, we refer to the main manuscript.

Method

In addition to the simulations in the main manuscript that differed on effect size (0.2 SD, 0.5 SD, and 0 SD), we now included a set of simulations with variable effect size. For these simulations, every participant's effect size was drawn from a population distribution with a variable group mean. The group mean was drawn from a normal distribution with a mean of 0.2 SD and a standard deviation of 0.1 SD. This results in individual effect sizes that get drawn from distributions with means that range from -0.1 SD to 0.5 SD.

In addition, we replicated all simulations reported in the main manuscript and the simulation above for the scenario where the standard deviation in the experimental group is 1.5 times larger than the standard deviation in the control group. This set-up reflects the scenario that there is more variability among participants in the experimental group than in the control group.

Our results are summarized in the same way as in the main manuscript, which results in 10 individual Figs.

Results

The Bayes factor results for the variable effect size simulations are shown in Fig S1. In all panels, the y-axis plots the Bayes factor in favor of the alternative hypothesis on a log scale. Different panels indicate different number of trials, and different columns indicate a different number of participants. The box-plots contain the middle 50% of simulation results, with the tails extending to 100% of the simulation results. The horizontal dashed line represents the case where evidence equally favors the alternative and the null hypothesis, results above the line favor the alternative hypothesis, and results below the line favor the null hypothesis.

S1 Fig about here

Comparing these results to those obtained in Fig 1 of the main manuscript shows that barring some minor simulation fluctuations, the results are functionally identical.

The proportion of 500 simulations for which the Bayes factor is lower than a certain cut-off value for median effect size is displayed in Fig S2. In all panels, the y-axis plots the proportion of 500 simulations for which the Bayes factor is lower than a certain cut-off value. Different panels indicate different number of trials, different columns indicate different number of participants, and different colors indicate different cut-off values.

S2 Fig about here

Again, comparing these results to those obtained in Fig 2 of the main manuscript shows that barring some minor simulation fluctuations, the results are functionally identical. These results demonstrate that individual differences in the mean of the underlying effect size distribution do not affect the outcome of these simulations.

The median Bayes factor results for the small effect size simulations with non-equal group standard deviations are shown in Fig S3. The layout is similar to that of Fig S1.

S3 Fig about here

Comparing these results to those obtained in Fig 1 of the main manuscript shows that the median Bayes factors are slightly lower for increased standard deviations in the experimental group. The qualitative pattern is identical to that reported in Fig 1.

The proportion of 500 simulations for which the Bayes factor is lower than a certain cut-off value for small effect size simulations with non-equal group standard deviations is displayed in Fig S4. The layout is similar to that of Fig S2. Comparing the results to those obtained in Fig 2 of the main manuscript mirrors our conclusion for the median Bayes factors: the higher standard deviation in the experimental group leads to lower Bayes factors across the board, but the qualitative pattern remains.

S4 Fig about here

The median Bayes factor results of the medium effect size simulations with non-equal group standard deviations are shown in Fig S5. The layout is similar to that of Figs S1 and S3.

S5 Fig about here

The proportion of 500 simulations for which the Bayes factor is lower than a certain cut-off value for medium effect size simulations with non-equal group standard deviations is displayed in Fig S6. The layout is similar to that of Fig S2 and S4. Comparing these results to those obtained in Figs 3 and 4 of the main manuscript shows a similar pattern to that obtained in the previous simulation set: an increased standard deviation in the experimental group leads to somewhat lower Bayes factors, but does not affect the qualitative pattern of results.

S6 Fig about here

The median Bayes factor results of the zero effect size simulations with non-equal group standard deviations are shown in Fig S7. The layout is similar to that of Figs S1, S3, and S5.

S7 Fig about here

The proportion of 500 simulations for which the Bayes factor is lower than a certain cut-off value for zero effect size simulations with non-equal group standard deviations is displayed in Fig S8. The layout is similar to that of Fig S2, S4, and S6. Comparing these results to those obtained in Figs 5 and 6 of the main manuscript shows results that are identical to those reported in the main manuscript, barring some sample fluctuations.

S8 Fig about here

The median Bayes factor results for the variable effect size simulations with non-equal group standard deviations are shown in Fig S9. The layout is similar to that of Figs S1, S3, S5, and S7.

S9 Fig about here

The proportion of 500 simulations for which the Bayes factor is lower than a certain cut-off value for variable effect size simulations with non-equal group standard deviations is displayed in Fig S10. The layout is similar to that of Fig S2, S4, S6, and S8. Comparing these results to those obtained in Figs S1 and S2 shows that an increased standard deviation in the experimental group leads to somewhat lower Bayes factors, but does not affect the qualitative pattern of results.

S10 Fig S10 about here

When taken together, the results indicate that varying the underlying effect size distribution within groups does not affect the results reported in the main manuscript. A difference in variance between the control and placebo groups does not change the qualitative pattern of results either. The overall increase in variance

leads to slightly lower Bayes factors overall. Had we decreased the variance in the experimental group, we would have likely obtained slightly higher Bayes factors. Overall, our results are very much in line with those reported in [1].

References

[1] van Ravenzwaaij D, Ioannidis JPA. A simulation study of the strength of evidence in the endorsement of medications based on two trials with statistically significant results. Manuscript submitted for publication.
